# Population pharmacokinetics of artesunate and dihydroartemisinin in pregnant and non-pregnant women with uncomplicated *Plasmodium falciparum* malaria in Burkina Faso: an open label trial

**DOI:** 10.12688/wellcomeopenres.14849.2

**Published:** 2020-01-10

**Authors:** Sofia Birgersson, Innocent Valea, Halidou Tinto, Maminata Traore-Coulibaly, Laeticia C. Toe, Richard M. Hoglund, Jean-Pierre Van Geertruyden, Stephen A. Ward, Umberto D’Alessandro, Angela Abelö, Joel Tarning

**Affiliations:** 1 Department of Pharmacology, University of Gothenburg, Gothenburg, 405 30, Sweden; 2 Institut de Recherche en Sciences de la Santé, Direction Régionale de l’Ouest Bobo-Dioulasso, Bobo-Dioulasso, Burkina Faso; 3 Institut de Recherche en Sciences de la Sante´, Unite´ de Recherche Clinique de Nanoro, Nanoro, Burkina Faso; 4 Department of Food Safety, Quality and Health, Faculty of Bioscience Engineering, Ghent University, Ghent, B-9000, Belgium; 5 Mahidol-Oxford Tropical Medicine Resarch Unit, Faculty of Tropical Medicine, Mahidol University, Bangkok, 10400, Thailand; 6 Nuffield Department of Medicine, University of Oxford, Oxford, OX3 7FZ, UK; 7 Global Health Institute, University of Antwerp, Antwerp, 2000, Belgium; 8 Department of Parasitology, Liverpool School of Tropical Medicine, Liverpool, L3 5QA, UK; 9 MRC Unit, London School of Hygiene & Tropical Medicine, Fajara, The Gambia

**Keywords:** Artemisinin-based combination therapy, Artesunate, Dihydroartemisinin, Mefloquine, Pregnancy, Malaria, Population pharmacokinetics, NONMEM

## Abstract

**Background**: Malaria during pregnancy is a major health risk for both the mother and the foetus. Pregnancy has been shown to influence the pharmacokinetics of a number of different antimalarial drugs. This might lead to an under-exposure in these patients which could increase the risk of treatment failure and the development of drug resistance. The study aim was to evaluate the pharmacokinetics of artesunate and dihydroartemisinin in pregnant and non-pregnant patients using a population modelling approach.

**Methods**: Twenty-four women in their second and third trimester of pregnancy and twenty-four paired non-pregnant women, all with uncomplicated *P. falciparum* malaria, were enrolled in this study. Treatment was a fixed-dose combination of oral artesunate and mefloquine once daily for three days. Frequent blood samples were collected and concentration-time data for artesunate and dihydroartemisinin were analysed simultaneously using nonlinear mixed-effects modelling.

**Results**: Artesunate pharmacokinetics was best described by a transit-compartment absorption model followed by a one-compartment disposition model under the assumption of complete *in vivo* conversion of artesunate into dihydroartemisinin. Dihydroartemisinin pharmacokinetics was best described by a one-compartment disposition model with first-order elimination. Pregnant women had a 21% higher elimination clearance of dihydroartemisinin, compared to non-pregnant women, resulting in proportionally lower drug exposure. In addition, initial parasitaemia and liver enzyme levels (alanine aminotransferase) were found to affect the relative bioavailability of artesunate.

**Conclusions**: Results presented here show a substantially lower drug exposure to the antimalarial drug dihydroartemisinin during pregnancy after standard oral treatment of artesunate and mefloquine. This might result in an increased risk of treatment failure and drug resistance development, especially in low transmission settings where relative immunity is lower.

**Trial registration**: ClinicalTrials.gov NCT00701961 (19/06/2008)

## Introduction

Malaria infection during pregnancy has been associated with major adverse health outcomes for both the mother and the foetus. In 2007, there were an estimated 85 million pregnancies in areas of endemic *P. falciparum* malaria ^1^. Pregnant women are more likely to get bitten by the vector, and to develop severe malaria ^2, 3^. Malaria during pregnancy has also adverse consequences for the foetus resulting in an increased risk of intrauterine growth retardation, low birth weight, stillbirth, and infant morbidity and mortality ^4^. The World Health Organization (WHO) is today recommending artemisinin based combination therapy (ACT) as first line therapy of uncomplicated *P. falciparum* malaria. This recommendation includes pregnant women in their second and third trimester ^5, 6^. ACTs include an artemisinin derivative (i.e. artesunate, artemether, dihydroartemisinin) together with a longer acting drug (i.e. mefloquine, piperaquine, lumefantrine, amodiaquine) ^7^. The artemisinins are highly effective, resulting in rapid parasite clearance during the first days of treatment ^8^. The longer acting partner drugs are responsible for eliminating residual parasites to prevent recrudescent malaria.

Artesunate is rapidly converted by pre-systemic hydrolysis, systemic esterases and cytochrome P450 (CYP) 2A6 into its active metabolite dihydroartemisinin ^9, 10^. Dihydroartemisinin is metabolized into inactive metabolites by glucuronidation by UDP-glucuronosyltransferase (UGT) 1A9 and 2B7 ^11^.

Pregnancy-related changes in the exposure to artesunate and dihydroartemisinin have been studied previously ^12– 14^. Morris *et al.* found a pregnancy-related increase in dihydroartemisinin clearance after oral artesunate treatment, resulting in approximately 42% lower drug exposure. Kloprogge *et al.* studied both intravenous and oral doses of artesunate in pregnant and non-pregnant women. Intravenous administration of artesunate showed similar disposition pharmacokinetics in both pregnant and non-pregnant women. However, a 23% decreased bioavailability of artesunate was observed after oral dosing which was explained by an increased pre-systemic activity during pregnancy.

A few other antimalarial drugs (e.g. chloroquine, lumefantrine and dihydroartemisinin) have shown a lower drug exposure in pregnant women compared to non-pregnant women, resulting in an increased risk for treatment failure and resistance development ^15– 21^.

The main aim of the present study was to evaluate the population pharmacokinetics of artesunate and its active metabolite, dihydroartemisinin, in a comparative study in pregnant and non-pregnant patients with uncomplicated *P. falciparum* malaria in Burkina Faso.

## Methods

### Study design and ethical approval

The study was conducted at the Nanoro District Hospital, Nanoro in Burkina Faso from 7 September 2008 to 15 January 2009. Clinical details and results from a non-compartmental analysis has been published previously ^22^. The investigation was a non-randomised parallel open label trial in pregnant and non-pregnant women with uncomplicated *P. falciparum* mono-infection. The study was approved by the National Health Ethics Committee in Burkina Faso (014-2008/CE-CM). The study was registered at www.clinicaltrials.gov ( NCT00701961) on June 19 ^th^ 2008.

### Study subjects

Pregnant and non-pregnant women with uncomplicated *P. falciparum* malaria mono-infection (defined as <50 000 parasites/µL and with no danger signs of severe malaria) were identified in two health facilities, the Centre de Sante´ et de Promotion Sociale (CSPS) of Nazoanga and of Nanoro, Burkina Faso. Non-pregnant women were selected to match the recruited pregnant women by age (either less or more than 20 years old) provided that they were residing in the same village as the pregnant women. Inclusion criteria for the study were; gestational age of more than 12 weeks, *P. falciparum* infection with a parasite density of less than 50,000 parasites/µL, willingness to sign or thumb print the written consent form, willingness to stay in the hospital for three days and to return for regular follow-up visits until delivery for treatment and observation, willingness to deliver at the health facility. A woman was excluded from the study if; she had a history of drug sensitivity to the studied drug or recent treatment with antimalarials or drugs known to interact with the studied drug, presence of any danger signs, physical findings of severe illness/severe anaemia, inability to tolerate oral medicine, chronic medical conditions requiring special care which could not be met by the study. Study procedures and objectives were explained in the local language by the study physician before obtaining a signed informed consent.

### Drug regimen and blood sampling

Treatment comprised a fixed-dose of artesunate/mefloquine-containing tablets (100 mg of artesunate and 220 mg of mefloquine) provided by Farmaguinhos, Rio de Janeiro, Brazil. A target daily dose of 8 mg/kg/day of mefloquine and 3.6 mg/kg/day of artesunate were given for three days to each patient. Number of tablets received was planned according to standard dosing according to body weight; women weighing <50 kg, 50–60 kg and >60 kg received a daily dose of 1.5, 2 and 2.5 tablets, respectively. Treatment was supervised and given after food.

Blood samples (2 mL) for pharmacokinetic analysis of artesunate and dihydroartemisinin were obtained by venous puncture or via a three-way tap attached to a catheter. Samples were collected before treatment and during the first day of treatment at time points: 0.25, 0.5, 1, 2, 3, 4, 5, 6, 8, 10 and 12 hours after dose. All samples were placed on ice immediately after blood collection and processed within 15 minutes. Plasma samples were obtained after centrifugation at 1,500 *g* for 10 minutes at 4°C and stored in liquid nitrogen until shipment on dry ice to the Department of Clinical Pharmacology, MORU, Bangkok, Thailand, for measurement of drug concentrations.

### Drug analysis

Samples were extracted by solid phase extraction and quantified using a validated liquid chromatography tandem mass spectrometry (LC-MS/MS) method ^23^. Briefly, artesunate and dihydroartemisinin standards and isotope-labelled internal standards were provided by the WorldWide Antimalarial Resistance Network (WWARN). Separation was performed using an Agilent 1200 system consisting of a binary LC pump, a vacuum degasser, a temperature-controlled micro wellplate autosampler set at 4°C and a column compartment set at 40°C (Agilent technologies, Santa Clara, USA). Quantification was performed using an API 5000 triple quadrupole mass spectrometer (Applied Biosystems/MDS SCIEX, Foster City, USA), with a TurboVTM ionization source (TIS) interface operated in the positive ion mode.

Data acquisition was performed using Analyst 1.5 (Applied Biosystems/MDS Sciex, Foster City, CA, USA). The lower limit of quantification (LLOQ) was 1.2 ng/mL and 2.0 ng/mL for artesunate and dihydroartemisinin, respectively. The total assay coefficient of variation was less than 7% for all quality control samples of artesunate (i.e. 2.90, 51.7 and 546 ng/mL, <3.5%) and dihydroartemisinin (i.e. 5.87, 117 and 1880 ng/mL, <6.4%) in this study. The laboratory participates in the WorldWide Antimalarial Resistance Network (WWARN) quality control and assurance proficiency testing program with satisfactory performance ^24^. Pharmacokinetic results of mefloquine will be reported elsewhere.

### Pharmacokinetic analysis

The population pharmacokinetic properties of artesunate and dihydroartemisinin were analysed using nonlinear mixed-effects modelling of the logarithmic plasma concentrations ( NONMEM version 7.1.2; ICON Development Solutions, MD). Pearl-Speaks-NONMEM (PsN; version 3.4.2), Pirana (version 2.4.0) and Xpose (version 4.0) package in R (version 2.13.1; The R Foundation for Statistical Computing) were used for post processing of the results and for automation of the modelling process ^25^.

Artesunate and dihydroartemisinin were modelled simultaneously and complete conversion of artesunate into its metabolite, dihydroartemisinin, was assumed ^9, 26^. The first-order conditional estimation (FOCE) method with interactions was used in the model building process. Data below the LLOQ was modelled as missing data (M1), categorical data (M3) or fixed to half of the LLOQ (M5) ^27^. When censored data were implemented with the M3-method a Laplacian estimation method was used.

Objective function value (OFV; proportional to minus twice the log likelihood of the observed data) and goodness-of-fit were used for model discrimination and graphical analysis, respectively. A drop in OFV of 3.84 was considered a significant (p=0.05) improvement in model fit when comparing two hierarchical models (one degree of freedom difference). One- two- and three-compartment models with first-order elimination from the central compartment were fitted to the plasma concentration-time data. Several different absorption models were evaluated (zero-order, first-order, absorption lag-time, sequential absorption, and a flexible transit compartment model with 1–10 fixed transit compartments). Inter-individual variability was evaluated exponentially on all parameters in the model ( Eq. 1).

$$\theta_{i}=\theta_{TV}\times \exp(\eta_{i})\;(\text{Eq}.1)$$

where θ _i_ is the individual parameter for the *i ^th^* patient and θ _TV_ is the typical value for parameter θ. η _i_ is the inter-individual variability for parameter θ, assumed to be normally distributed with mean zero and variance ω ^2^. Relative bioavailability of artesunate was evaluated by fixing the population value to unity and estimating the inter-individual variability.

The residual random variability was modelled with two separate additive error models (i.e. artesunate and dihydroartemisinin) on the log-transformed drug concentrations, being essentially equivalent to an exponential residual error on an arithmetic scale.

Body weight was evaluated as an allometric function on all clearance and volume parameters. The allometrically scaled parameters were centered on the median body weight of the studied population and scaled to a power of 0.75 and 1 for clearance and volume parameters, respectively.

Stepwise forward inclusion ( *p*<0.05) was used for all other continuous and categorical covariates followed by a stepwise backward exclusion ( *p*<0.01). Parasite biomass, alanine aminotransferase (ALT), aspartate aminotransferase (AST), bilirubin levels, and haemoglobin levels at enrolment were tested as continuous covariates while pregnancy was evaluated as both a continuous (estimated gestational age; 0–8 months) and a categorical covariate (pregnant vs non-pregnant).

A categorical pregnancy effect was evaluated using a full covariate approach (i.e. the pregnancy effect was added simultaneously on relative oral bioavailability, all clearance parameters and on mean transit time). The full covariate model was bootstrapped (n=500) and the 80% confidence interval of the covariate effect on each of the pharmacokinetic parameters visualized to investigate the clinical impact and the predicted variability of the covariate effect.

Basic graphical goodness-of-fit was evaluated by plotting observed artesunate and dihydroartemisinin concentrations against individually and population predicted concentrations and by plotting conditionally weighted residuals against population predicted concentrations and time. Eta and epsilon shrinkages were also evaluated to assess reliability of goodness-of-fit characteristics ^28^. Prediction-correct visual predictive checks were performed using 2,000 simulations. Bootstrap diagnostics stratified on pregnancy, were performed using 1,000 re-sampled datasets to obtain standard errors for parameter estimates and non-parametric confidence intervals to evaluate parameter precision.

The final model was also used to simulate pregnant (n = 1,000) and non-pregnant (n = 1,000) women in order to investigate the differences in secondary exposure parameters (AUC and C _MAX_) associated with pregnancy *.* Simulated pregnant and non-pregnant women were identical with respect to body weight and other co-variates, except pregnancy. All individual exposure parameters were divided on the average value for a non-pregnant women, and presented as a box-plot to illustrate trends and variabilities.

## Results

48 women (24 pregnant and 24 non-pregnant patients) completed the pharmacokinetic study (one pregnant woman was lost to follow-up due to moving out of the study area). No adverse effects, related to study treatment, were observed and treatment efficacy was excellent in both groups with total parasite clearance by day three ^22^. Consort checklist and flow-chart are available from figshare ^29^). Parasites were detected in one pregnant woman at a revisit at day 49, classified as a new infection by genotyping. No recrudescent malaria was observed in any of the patients.

Demographic and clinical data are presented in Table 1. In both groups age, weight and height were comparable, but a higher parasite density and a significantly lower haemoglobin level were found in the pregnant women. The frequent sampling design resulted in full pharmacokinetic concentration-time profiles for artesunate and dihydroartemisinin in both groups. Artesunate was rapidly converted into dihydroartemisinin, and artesunate concentrations reached the LLOQ within six hours in all patients. 62% of the artesunate samples, and 21% of the dihydroartemisinin samples were measured to be below the LLOQ.

**Table 1.** Admission demographics of study population.

Parameter	Pregnant women median (range)	Non-pregnant women median (range)
Number of patients	24	24
Age (year)	20.5 (18–39)	25 (18–48)
Weight (kg)	52 (46–70)	53 (45–70)
Height (cm)	160 (150–170)	160 (160–170)
Parasite density (count/µL)	810 (79–54,000)	240 (18–2,444)
Haemoglobin (g/dL)	9.10 (7.1–11)	12 (7.8–14)
AST (units/L)	20 (3.2–84)	27.9 (8.2–59.8)
ALT (units/L)	20.3 (3.2–63)	21.75 (9.9–61.2)
Bilirubin (mg/dL)	0.45 (0.2–1.9)	0.5 (0.2–2)
Gestational age (month)		
Second trimester, n=12	5 (4–5)	0
Third trimester, n=12	7 (6–8)	0

AST, aspartate aminotransferase; ALT, aspartate aminotransferase

### Pharmacokinetic analysis

**Figure 1.**
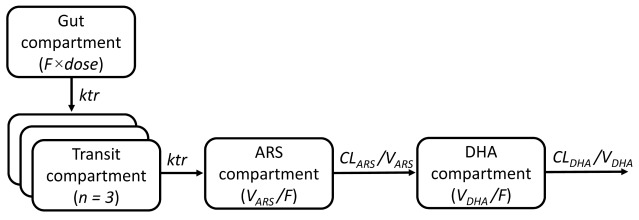
Structural representation of the final population pharmacokinetic model. *ARS* is artesunate; *DHA* is dihydroartemisinin; *k _TR_* is the absorption rate constant; *CL/F* is the apparent elimination clearance; *V/F* is the apparent volume of distribution; *F* is the relative bioavailability of artesunate.

**Figure 2.**
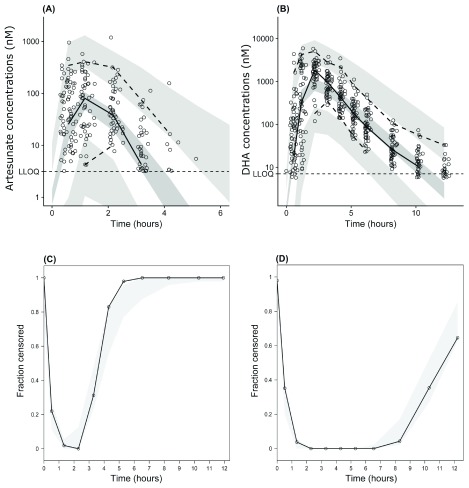
Prediction-corrected visual predictive check of the final population pharmacokinetic model. Predictive performance of the final population pharmacokinetic model of artesunate ( **A**) and dihydroartemisinin ( **B**) in pregnant and non-pregnant women. Open circles (upper panel) represent the observed concentrations and the solid lines represent the 5 ^th^, 50 ^th^ and 95 ^th^ percentiles of the observed concentrations. Shaded areas show the 95% confidence interval of the 5 ^th^, 50 ^th^ and 95 ^th^ percentiles of simulated concentrations. Solid black lines (lower panel) represent the observed fraction of data below the lower limit of quantification and the shaded area represents the 95% confidence intervals of simulated data below the lower limit of quantification for artesunate ( **C**) and dihydroartemisinin ( **D**).

The pharmacokinetic properties of artesunate and dihydroartemisinin were described simultaneously in a drug-metabolite model, assuming complete *in vivo* conversion of artesunate into dihydroartemisinin ( Figure 1). A transit-compartment (n = 3) absorption model for artesunate was superior to all other absorption models tested (ΔOFV > 242). Allowing for inter-individual variability in the relative bioavailability of artesunate improved the model fit substantially (ΔOFV = 27). Artesunate disposition was defined by a central disposition compartment with no additional benefit of adding a peripheral disposition compartment (p > 0.05). Dihydroartemisinin was best described by a one-compartment disposition model with first-order elimination. However, an additional peripheral disposition compartment for dihydroartemisinin improved the model fit significantly (ΔOFV = 21), but resulted in an unrealistic terminal elimination half-life of 9 hours and was therefore not carried forward ^30– 33^. A third disposition compartment did not improve the model fit (p > 0.05). Ignoring data below the LLOQ (missing data) or imputing them as half the LLOQ resulted in model misspecifications due to the large fraction of censored data (results not shown). The best performing model was obtained when data below the LLOQ was modelled as categorical data (i.e. M3), thereby maximizing the probability that observations below the LLOQ are predicted to be below the LLOQ ( Figure 2). Inter-individual variability was retained on artesunate elimination clearance, dihydroartemisinin elimination clearance, mean–transit-time, and relative bioavailability. A separate additive error model for artesunate and dihydroartemisinin, respectively, described the random residual variability adequately.

Body weight implemented as an allometric function on clearance and volume parameters, improved the model fit (ΔOFV = 6.3) and was retained in the final model due to the strong biological prior of this covariate, and to remove the potential bias of systematically different body weights between pregnant and non-pregnant women. Pregnancy as a categorical covariate had a significant (ΔOFV = 20) impact on oral clearance of dihydroartemisinin, resulting in a 21% increased elimination clearance of dihydroartemisinin in pregnant women as compared to non-pregnant women. In addition, parasite biomass (ΔOFV = 10) and ALT (ΔOFV = 14) at enrolment were found to be significant covariates on the relative bioavailability; 14% increase in relative bioavailability per each increase in the natural logarithm of the parasite biomass, and 2.2% increase in relative bioavailability per each unit (IU) increase in ALT).

**Figure 3.**
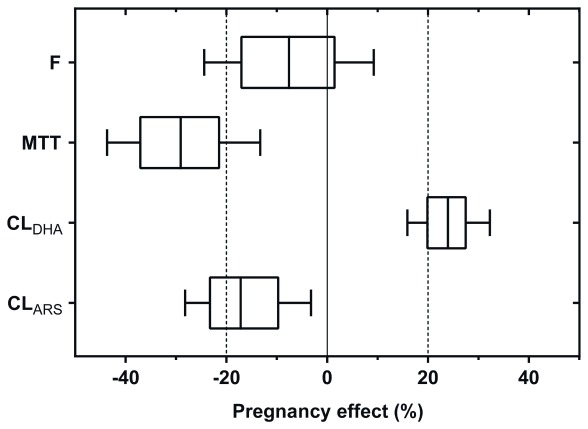
The impact of pregnancy on primary pharmacokinetic parameters. Box and whisker plot of the results from the full covariate model investigating pregnancy as a categorical covariate. Boxes represent the 25 ^th^ to 75 ^th^ percentiles and whiskers represent the 10 ^th^ to 90 ^th^ percentiles. The solid vertical line represents no covariate effect and the dashed vertical lines represent a covariate effect of ±20%, which is assumed to be associated with clinical significance. *F* is the relative oral bioavailability, *CL _ARS_/F* is the apparent elimination clearance of artesunate, *CL _DHA_/F* is the apparent elimination clearance of dihydroartemisinin, and *MTT* is the mean absorption transit time. The covariate was added as a categorical function and bootstrapped (n=500).

Final parameter estimates are summarized in Table 2. The full covariate approach investigating the impact of pregnancy confirmed the above covariate relationship, resulting in an increased dihydroartemisinin elimination clearance of 24% in pregnant women, causing a proportional decrease in total drug exposure to dihydroartemisinin ( Figure 3).

**Table 2.** Final model parameters describing artesunate and dihydroartemisinin population pharmacokinetics in pregnant and non-pregnant women.

Parameters	Population estimate (RSE%)	CI. 95%	IIV CV% (RSE%)	CI. 95%
F (%)	100 *fixed*	-	30.5 (20.0)	16.8-38.7
Nr. of trans comp	3 *fixed*	-	-	-
MTT (h)	0.832 (8.56)	0.695-0.979	61.4 (12.8)	46.2-76.0
CL _ARS_/F (L/h)	3,570 (9.22)	2,990-4,290	26.4 (27.5)	5.47-35.2
V _ARS_/F (L)	1,700 (11.2)	1,370-2,110	-	-
CL _DHA_/F (L/h)	190 (5.87)	168-213	9.00 (23.7)	3.63-11.6
V _DHA_/F (L)	267 (6.49)	236-301	-	-
PREG _CL_DHA_ (%)	21.4 (16.3)	14.3-27.9	-	-
ALT _F_ (%)	2.15 (29.2)	1.10-3.57		
Biomass _F_ (%)	13.8 (23.6)	7.32-20.0		
σ _ARS_	0.892 (11.5)	0.707-1.10	-	-
σ _DHA_	0.660 (9.84)	0.534-0.780	-	-

*ARS, artesunate; DHA, dihydroartemisinin; CL/F,* apparent elimination clearance; *V/F*, apparent volume of distribution; *MTT*, mean transit time of the absorption phase; *F*, relative oral bioavailability; *Nr. trans comp*, number of transit compartments in the absorption model; PREG _CL_DHA_, proportional increase in CL _DHA_/F with pregnancy; ALT _F_, linear increase in F with ALT; Biomass _F_, linear increase in F with parasite biomass at enrolment; *σ*, additive residual error as variance. RSE is the relative standard error calculated as 100 *x standard deviation/mean*. CV% is the coefficient of variation calculated as $100\times \sqrt{(e^{variance}-1})$ for inter-individual variability (IIV). Population parameter and IIV estimates are estimated directly by NONMEM. RSE% and 95% confidence intervals (CI. 95%) are based on 860 successful bootstrap runs (out of 1,000).

**Figure 4.**
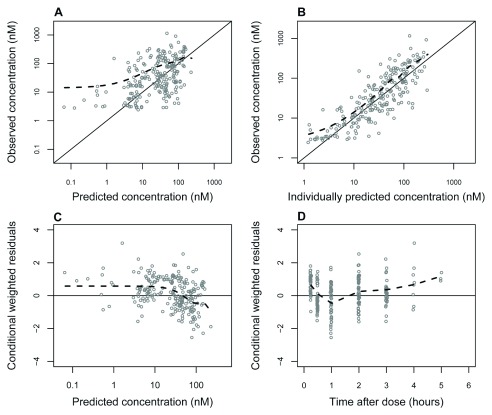
Goodness-of-fit diagnostics of the final population pharmacokinetic model for artesunate. Descriptive performance of the final population pharmacokinetic model in pregnant and non-pregnant women. Lines represent weighted least-squares regression (dashed) and lines of identity (solid).

**Figure 5.**
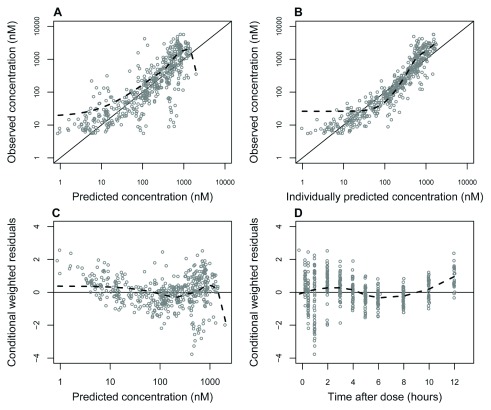
Goodness-of-fit diagnostics of the final population pharmacokinetic model for dihydroartemisinin. Descriptive performance of the final population pharmacokinetic model in pregnant and non-pregnant women. Lines represent weighted least-squares regression (dashed) and lines of identity (solid).

**Figure 6.**
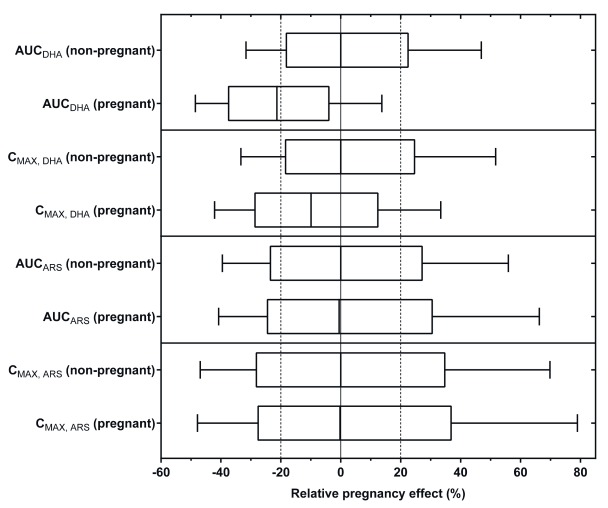
The impact of pregnancy on secondary pharmacokinetic parameters. Relative effect of pregnancy on simulated exposure (AUC and C _MAX_) to artesunate and dihydroartemisinin. The final model was used to simulate 1,000 non-pregnant and pregnant woman after an oral single dose of artesunate. All individual exposure parameters were divided on the average value for a non-pregnant women, to generate relative pregnancy effects. Boxes represent the 25 ^th^ to 75 ^th^ percentiles and whiskers represent the 10 ^th^ to 90 ^th^ percentiles. The vertical black line represents no covariate effect and the dashed vertical lines represent a covariate effect of ±20%, which is assumed to be associated with clinical significance.

The final model showed satisfactory goodness-of-fit diagnostics for both artesunate and dihydroartemisinin ( Figure 4 and Figure 5) and predictive performance ( Figure 2). The pharmacometric model code is available in Table 3. Calculated epsilon-shrinkage was low (11.1% and 10.4% for artesunate and dihydroartemisinin, respectively) indicating that model diagnostics can be assessed reliably. However, the estimated eta shrinkage was relatively high for clearance parameters (i.e. artesunate clearance = 30%, dihydroartemisinin clearance = 40%, mean transit time = 5.7%, bioavailability = 25%) and empirical Bayes estimates should therefore be interpreted with caution ^28^. Simulations of the exposure to artesunate and dihydroartemisinin in pregnant and non-pregnant women can be seen in Figure 6.

**Table 3.** Final population pharmacokinetic model of artesunate and dihydroartemisinin in pregnant and non-pregnant women with uncomplicated *falciparum* malaria.

$INPUT ID ; Patient ID TIME ; Time of sample DV ; Dependent variable (natural logarithm of observed concentrations) WT ; Body weight (covariate) EVID ; Event ID record MDV ; Missing dependent variable (1=missing) AMT ; Dose amount CMT ; Compartment (1=dose, 2=artesunate, 3=dihydroartemisinin) BQL ; Below quantification limit (1=below BQL) PREG ; Pregnancy (covariate; 0=non-pregnant, 1=pregnant) LNPC ; Parasite count (covariate; natural logarithm of parasite count) HB ; Haemoglobin measurement (covariate) AST ; AST (covariate) ALT ; ALT (covariate) BIL ; Bilirubin (covariate) EGA ; Estimated gestational age (covariate) $DATA dataset.csv IGNORE=# $SUBROUTINE ADVAN5 TRANS1 $MODEL COMP = (1) ; Dose COMP = (2) ; Artesunate COMP = (3) ; Dihydroartemisinin COMP = (4) ; Transit compartment 1 COMP = (5) ; Transit compartment 2 COMP = (6) ; Transit compartment 3 $PK ;------------------------------Pregnancy covariate---------------------------------------------------------------- PREGNANCY = (1 + THETA(7) * PREG) ; Linear covariate relationship for pregnancy ;------------------------------------------------------------------------------------------------------------------- ;------------------------------Liver enzyme level covariate---------------------------------------------------------------- LIVER = (1 + THETA(8) * (ALT - 20.75)) ; Linear covariate relationship for liver enzyme ;------------------------------------------------------------------------------------------------------------------- ;------------------------------Parasite biomass covariate----------------------------------------------------------- PARASITE = (1 + THETA(9) * (LNPC - 5.88)) ; Linear covariate relationship for parasite biomass ;-------------------------------------------------------------------------------------------------------------------- TVCLP = THETA(1) * ((WT/52)**0.75) ; Population artesunate clearance CLP = TVCLP * EXP(ETA(1)) ; Individual artesunate clearance TVV2 = THETA(2) * ((WT/52)**1) ; Population artesunate volume V2 = TVV2 * EXP(ETA(2)) ; Individual artesunate volume TVCLM = THETA(3) * ((WT/52)**0.75) * PREGNANCY ; Population DHA clearance CLM = TVCLM * EXP(ETA(3)) ; Individual DHA clearance TVV3 = THETA(4) * ((WT/52)**1) ; Population DHA volume V3 = TVV3 * EXP(ETA(4)) ; Individual DHA volume TVMT = THETA(5) ; Population mean transit time MT = TVMT * EXP(ETA(5)) ; Individual mean transit time TVF1 = THETA(6) * LIVER * PARASITE ; Population relative bioavailability F1 = TVF1 * EXP(ETA(6)) ; Individual relative bioavailability NN = 3 ; Number of transit compartments KTR = (NN + 1) / MT ; Transit rate constant K14 = KTR ; Transit rate between compartment 1 and 4 K45 = KTR ; Transit rate between compartment 4 and 5 K56 = KTR ; Transit rate between compartment 5 and 6 K62 = KTR ; Transit rate between compartment 6 and 2 K23 = CLP / V2 ; Elimination of artesunate K30 = CLM / V3 ; Elimination of dihydroartemisinin $ERROR IF(CMT.EQ.2) THEN IPRED = A(2) / V2 ; Predicted plasma concentration of artesunate W = SQRT(SIGMA(1,1)) ; Residual error artesunate ENDIF IF(CMT.EQ.3) THEN IPRED = A(3) / V3 ; Predicted plasma concentration of dihydroartemisinin W = SQRT(SIGMA(2,2)) ; Residual error dihydroartemisinin ENDIF IF(IPRED.GT.0) IPRED = LOG(IPRED) ; Natural logarithm of predictions ;-------------------------------M3-method for incorporating concentratoins below LLOQ---------------------- DUM = (LLOQ-IPRED) / W CUMD = PHI(DUM) ;---------------------------------------------------------------------------------------------------------------- ; -----------------------------------------Prediction DV>=LLOQ----------------------------------------------- IRES = IPRED - DV IWRES = IRES / W IF(BQL.EQ.0.AND.CMT.EQ.2) THEN ; Calculating Y for artesunate F_FLAG = 0 Y = IPRED + ERR(1) ENDIF IF(BQL.EQ.0.AND.CMT.EQ.3) THEN ; Calculating Y for dihydroartemisinin F_FLAG = 0 Y = IPRED + ERR(2) ENDIF ;---------------------------------------------------------------------------------------------------------------- ;--------------------------------------------- Likelihood DV<LLOQ------------------------------------------- IF(BQL.EQ.1) THEN ; Calculating Y for data below LLOQ F_FLAG = 1 Y = CUMD + 0.000001 ENDIF ;---------------------------------------------------------------------------------------------------------------- $THETA ; Initial estimates of theta (0, 3570) ; 1. Artesunate clearance (0, 1700) ; 2. Artesunate volume of distribution (0, 190) ; 3. Dihydroartemisinin clearance (0, 267) ; 4. Dihydroartemisinin volume of distribution (0, 0.832) ; 5. Mean transit time (1 FIX) ; 6. Relative bioavailability (-1, 0.214) ; 7. Pregnancy effect on dihydroartemisinin clearance (-0.024, 0.0215, 0.057) ; 8. Liver enzyme effect on relative bioavailability (-0.199, 0.138, 0.334) ; 9. Parasite biomass effect on relative bioavailability $OMEGA ; Initial estimates for omega (0.0672) ; 1. IIV artesunate clearance (0 FIX) ; 2. IIV artesunate volume of distribution (0.00810) ; 3. IIV dihydroartemisinin clearance (0 FIX) ; 4. IIV dihydroartemisinin volume of distribution (0.320) ; 5. IIV mean transit time (0.0887) ; 6. IIV relative bioavailability $SIGMA ; Initial estimates of sigma (0.892) ; Residual variability artesunate (0.660) ; Residual variability dihydroartemisinin

## Discussion

In this study, a population pharmacokinetic model was developed for artesunate and dihydroartemisinin in pregnant and non-pregnant women in Burkina Faso, treated with a fixed-dose combination of artesunate and mefloquine. The final model was a one-compartment disposition model for both artesunate and its active metabolite, dihydroartemisinin. Artesunate absorption was described with a flexible transit compartment model allowing for inter-individual variability in the relative bioavailability. Pregnancy had a significant impact on the elimination clearance of dihydroartemisinin, resulting in a 21% reduced drug exposure compared to non-pregnant women.

Artesunate and dihydroartemisinin population pharmacokinetics have been described previously with one-compartment disposition models in pregnant and non-pregnant women after oral administration, supporting the present findings ^12, 31^. Artesunate absorption is known to be rapid and was best described by a transit compartment model (three transit compartments), which is in agreement with a previously published population pharmacokinetic model in pregnant women by *Kloprogge et al.* Furthermore, the transit absorption model offers a biologically plausible parameterization of drugs that show varying degrees of lag-time absorption. The pregnancy-related alterations on the pharmacokinetic properties of the artemisinins derivatives are also supported in literature. Morris *et al.* found a pregnancy-induced increase of 42% in the elimination clearance of dihydroartemisinin ^12^. In the study by Tarning *et al.* ^31^, where oral dihydroartemisinin was administered alone, pregnancy was found to influence the bioavailability of dihydroartemisinin resulting in a 38% reduction in the exposure in pregnant compared to non-pregnant women. The present analysis also identified parasite biomass as a covariate on the relative bioavailability, resulting in an increased exposure to artesunate and dihydroartemisinin in patients with higher initial parasitaemia. This covariate effect has been identified also in another study ^14^. This previously published clinical study showed opposite pharmacokinetic effects of malaria (87% increase) and pregnancy (23% decrease) on the absolute oral bioavailability of artesunate ^13, 14^. A sequential intravenous and oral dosing regimen in pregnant and post-partum women during the acute malaria phase and at recovery (i.e. healthy) enabled these covariate effects to be dissected and quantified. The results in the present study support these findings. In addition to the disease effect, liver enzyme levels (ALT) was found to affect the relative bioavailability, resulting in an increased exposure with increasing ALT levels. This is likely to be explained by a decreased first-pass metabolism of artesunate.

The full covariate approach supported the step-wise covariate results, demonstrating a mean increased dihydroartemisinin clearance of 24% in pregnant women compared to non-pregnant women. There was also a trend towards a decreased mean transit absorption time in pregnant women. This suggests that an additional pregnancy effect in the absorption phase might be significant in a larger patient study. There was also a trend of decreasing artesunate clearance in pregnant women but it could not be substantiated by the step-wise covariate results.

The non-compartmental analysis of this clinical study by Valea *et al.*, demonstrated an increased exposure to artesunate in pregnant women, resulting from an unexplained decrease in artesunate elimination clearance ^22^. This decrease was considered highly contradictive since elimination generally increases during pregnancy due to induced enzyme systems ^34^. The increased artesunate exposure could not be explained by a model-independent analysis. Also, no significant differences were found in the exposure to dihydroartemisinin in the model-independent analysis, but large variability was noted and thus limited power to detect differences. In the present paper, a model based approach was implemented for a more mechanistic understanding of the impact of pregnancy on the pharmacokinetics of artesunate and dihydroartemisinin. In contradiction to the NCA analysis, no pregnancy related effect was found on clearance for artesunate although a trend towards a decreased clearance was seen in the full covariate model.

It is well known that haemoglobin levels are decreased in pregnant women due to the increased need of iron for the mother and the foetus ^35^. Indeed, haemoglobin levels were significantly lower in the pregnant group in this study. However, haemoglobin was not a significant covariate in the present study when pregnant and non-pregnant women were modelled simultaneously and separately.

In conclusion, a population pharmacokinetic model was developed for artesunate and dihydroartemisinin. A pregnancy related increase in the elimination of dihydroartemisinin was found, resulting in a proportionally decreased exposure to dihydroartemisinin. This could result in an increased risk of failure and possibly a need of increased dosing of artesunate during pregnancy; especially in low-transmission settings where the acquired immunity is relatively lower. However, the clinical relevance of a lower exposure needs to be further evaluated in prospective studies.

## Data availability

### Underlying data

Due to ethical and security considerations, the data that supports the findings in this study can be accessed only through the Data Access Committee at Mahidol Oxford Tropical Medicine Research Unit (MORU). The application form and data sharing policy can be found here: http://www.tropmedres.ac/data-sharing.

The full NONMEM model code describing the final population pharmacokinetic model is available in Table 3, and also in the open pharmacometric model repository hosted by DDMoRe.

NONMEM model code, Accession number DDMODEL00000297: http://repository.ddmore.foundation/model/DDMODEL00000297/

### Reporting guidelines

CONSORT checklist and flow diagram for “Population pharmacokinetics of artesunate and dihydroartemisinin in pregnant and non-pregnant women with uncomplicated Plasmodium falciparum malaria in Burkina Faso” https://doi.org/10.6084/m9.figshare.7699835.v1 ^29^.

Data are available under the terms of the Creative Commons Zero "No rights reserved" data waiver (CC0 1.0 Public domain dedication).

## Consent

The study was approved by the National Health Ethics Committee in Burkina Faso (014-2008/CE-CM). The study was registered at www.clinicaltrials.gov (NCT00701961). Study procedures and objectives were explained in the local language by the study physician before obtaining a signed informed consent.

## References

[1] Dellicour S Tatem AJ Guerra CA: Quantifying the number of pregnancies at risk of malaria in 2007: a demographic study. *PLoS Med.* 2010;7(1):e1000221. 10.1371/journal.pmed.1000221 20126256 PMC2811150

[2] Lindsay S Ansell J Selman C: Effect of pregnancy on exposure to malaria mosquitoes. *Lancet.* 2000;355(9219):1972. 10.1016/S0140-6736(00)02334-5 10859048

[3] World Health Organization: A Strategic Framework for Malaria Prevention and Control During Pregnancy in the African Region.2004 Reference Source

[4] Steketee RW Nahlen BL Parise ME: The burden of malaria in pregnancy in malaria-endemic areas. *Am J Trop Med Hyg.* 2001;64(1–2 Suppl):28–35. 10.4269/ajtmh.2001.64.28 11425175

[5] WHO: Guidelines for the Treatment of Malaria. World Health Organization;2010. 25473692

[6] Vallely A Vallely L Changalucha J: Intermittent preventive treatment for malaria in pregnancy in Africa: what’s new, what's needed? *Malar J.* 2007;6:16. 10.1186/1475-2875-6-16 17306014 PMC1805504

[7] World Health Organization: WHO | Guidelines for the treatment of malaria. Third edition. World Health Organization;2015. 26020088

[8] White NJ: Assessment of the pharmacodynamic properties of antimalarial drugs *in vivo.* *Antimicrob Agents Chemother.* 1997;41(7):1413–22. 10.1128/AAC.41.7.1413 9210658 PMC163932

[9] Li XQ Bjorkman A Andersson TB: Identification of human cytochrome P _450_s that metabolise anti-parasitic drugs and predictions of *in vivo* drug hepatic clearance from *in vitro* data. *Eur J Clin Pharmacol.* 2003;59(5–6):429–42. 10.1007/s00228-003-0636-9 12920490

[10] Navaratnam V Mansor SM Sit NW: Pharmacokinetics of artemisinin-type compounds. *Clin Pharmacokinet.* 2000;39(4):255–70. 10.2165/00003088-200039040-00002 11069212

[11] Ilett KF Ethell BT Maggs JL: Glucuronidation of dihydroartemisinin *in vivo* and by human liver microsomes and expressed UDP-glucuronosyltransferases. *Drug Metab Dispos.* 2002;30(9):1005–12. 10.1124/dmd.30.9.1005 12167566

[12] Morris CA Onyamboko MA Capparelli E: Population pharmacokinetics of artesunate and dihydroartemisinin in pregnant and non-pregnant women with malaria. *Malar J.*BioMed Central;2011;10:114. 10.1186/1475-2875-10-114 21548983 PMC3098207

[13] McGready R Phyo AP Rijken MJ: Artesunate/dihydroartemisinin pharmacokinetics in acute falciparum malaria in pregnancy: absorption, bioavailability, disposition and disease effects. *Br J Clin Pharmacol.* 2012;73(3):467–77. 10.1111/j.1365-2125.2011.04103.x 21950338 PMC3370352

[14] Kloprogge F McGready R Phyo AP: Opposite malaria and pregnancy effect on oral bioavailability of artesunate - a population pharmacokinetic evaluation. *Br J Clin Pharmacol.* 2015;80(4):642–53. 10.1111/bcp.12660 25877779 PMC4594700

[15] Ward SA Sevene EJ Hastings IM: Antimalarial drugs and pregnancy: safety, pharmacokinetics, and pharmacovigilance. *Lancet Infect Dis.* 2007;7(2):136–44. 10.1016/S1473-3099(07)70025-7 17251084

[16] Tarning J McGready R Lindegardh N: Population pharmacokinetics of lumefantrine in pregnant women treated with artemether-lumefantrine for uncomplicated *Plasmodium falciparum* malaria. *Antimicrob Agents Chemother.* 2009;53(9):3837–46. 10.1128/AAC.00195-09 19564366 PMC2737887

[17] McGready R Stepniewska K Ward SA: Pharmacokinetics of dihydroartemisinin following oral artesunate treatment of pregnant women with acute uncomplicated falciparum malaria. *Eur J Clin Pharmacol.* 2006;62(5):367–71. 10.1007/s00228-006-0118-y 16552504

[18] Karunajeewa HA Salman S Mueller I: Pharmacokinetics of chloroquine and monodesethylchloroquine in pregnancy. *Antimicrob Agents Chemother.* 2010;54(3):1186–92. 10.1128/AAC.01269-09 20086162 PMC2825967

[19] Dondorp AM Nosten F Yi P: Artemisinin resistance in *Plasmodium falciparum* malaria. *N Engl J Med.* 2009;361(5):455–67. 10.1056/NEJMoa0808859 19641202 PMC3495232

[20] Tarning J Kloprogge F Dhorda M: Pharmacokinetic properties of artemether, dihydroartemisinin, lumefantrine, and quinine in pregnant women with uncomplicated *plasmodium falciparum* malaria in Uganda. *Antimicrob Agents Chemother.* 2013;57(10):5096–103. 10.1128/AAC.00683-13 23917320 PMC3811434

[21] Kloprogge F Piola P Dhorda M: Population Pharmacokinetics of Lumefantrine in Pregnant and Nonpregnant Women With Uncomplicated *Plasmodium falciparum* Malaria in Uganda. *CPT pharmacometrics Syst Pharmacol.* 2013;2(11):e83. 10.1038/psp.2013.59 24226803 PMC3852159

[22] Valea I Tinto H Traore-Coulibaly M: Pharmacokinetics of co-formulated mefloquine and artesunate in pregnant and non-pregnant women with uncomplicated *Plasmodium falciparum* infection in Burkina Faso. *J Antimicrob Chemother.* 2014;69(9):2499–507. 10.1093/jac/dku154 24891429 PMC4130382

[23] Hanpithakpong W Kamanikom B Dondorp AM: A liquid chromatographic-tandem mass spectrometric method for determination of artesunate and its metabolite dihydroartemisinin in human plasma. *J Chromatogr B Analyt Technol Biomed Life Sci.* 2008;876(1):61–8. 10.1016/j.jchromb.2008.10.018 18990614

[24] Lourens C Lindegardh N Barnes KI: Benefits of a pharmacology antimalarial reference standard and proficiency testing program provided by the Worldwide Antimalarial Resistance Network (WWARN). *Antimicrob Agents Chemother.* 2014;58(7):3889–94. 10.1128/AAC.02362-14 24777099 PMC4068537

[25] Keizer RJ van Benten M Beijnen JH: Piraña and PCluster: a modeling environment and cluster infrastructure for NONMEM. *Comput Methods Programs Biomed.* 2011;101(1):72–9. 10.1016/j.cmpb.2010.04.018 20627442

[26] Teja-Isavadharm P Watt G Eamsila C: Comparative pharmacokinetics and effect kinetics of orally administered artesunate in healthy volunteers and patients with uncomplicated falciparum malaria. *Am J Trop Med Hyg.* 2001;65(6):717–21. 10.4269/ajtmh.2001.65.717 11791963

[27] Beal SL: Ways to fit a PK model with some data below the quantification limit. *J Pharmacokinet Pharmacodyn.* 2001;28(5):481–504. 10.1023/A:1012299115260 11768292

[28] Savic RM Karlsson MO: Importance of shrinkage in empirical bayes estimates for diagnostics: problems and solutions. *AAPS J.* 2009;11(3):558–69. 10.1208/s12248-009-9133-0 19649712 PMC2758126

[29] Tarning J: Supplementary files. *figshare.*Fileset.2019 10.6084/m9.figshare.7699835.v1

[30] Nosten F White NJ: Artemisinin-based combination treatment of falciparum malaria. *Am J Trop Med Hyg.* 2007;77(6 Suppl):181–92. 10.4269/ajtmh.2007.77.181 18165491

[31] Tarning J Rijken MJ McGready R: Population pharmacokinetics of dihydroartemisinin and piperaquine in pregnant and nonpregnant women with uncomplicated malaria. *Antimicrob Agents Chemother.* 2012;56(4):1997–2007. 10.1128/AAC.05756-11 22252822 PMC3318332

[32] Tarning J Kloprogge F Piola P: Population pharmacokinetics of Artemether and dihydroartemisinin in pregnant women with uncomplicated *Plasmodium falciparum* malaria in Uganda. *Malar J.* 2012;11:293. 10.1186/1475-2875-11-293 22913677 PMC3502166

[33] Ali S Najmi MH Tarning J: Pharmacokinetics of artemether and dihydroartemisinin in healthy Pakistani male volunteers treated with artemether-lumefantrine. *Malar J.* 2010;9:275. 10.1186/1475-2875-9-275 20932349 PMC2959074

[34] Anderson GD: Pregnancy-induced changes in pharmacokinetics: a mechanistic-based approach. *Clin Pharmacokinet.* 2005;44(10):989–1008. 10.2165/00003088-200544100-00001 16176115

[35] Peña-Rosas JP De-Regil LM Garcia-Casal MN: Daily oral iron supplementation during pregnancy. *Cochrane Database Syst Rev.* 2015; (7):CD004736. 10.1002/14651858.CD004736.pub5 26198451 PMC8918165

