# Supplementary material for: Population pharmacokinetics of artesunate and dihydroartemisinin in pregnant and non-pregnant women with uncomplicated Plasmodium falciparum malaria in Burkina Faso: an open label trial
Source: Wellcome Open Res. 2020 Jan 10;4:45. Originally published 2019 Mar 7. [Version 2] doi: 10.12688/wellcomeopenres.14849.2 (PMC6974929; doi:10.12688/wellcomeopenres.14849.2)
Supplement: Peer review history

# Peer review: Reviewer response for version 2

**DOI:** 10.21956/wellcomeopenres.17157.r37566

**Published:** 2020-01-21

**Authors:** Lawrence Fleckenstein

**Affiliations:** 1 College of Pharmacy, University of Iowa, Iowa City, IA, USA

I have reviewed the revisions made by Professor Tarning and he has satisfactorily responded to all of the points I made in my review.

I confirm that I have read this submission and believe that I have an appropriate level of expertise to confirm that it is of an acceptable scientific standard.

# Peer review: Reviewer response for version 1

**DOI:** 10.21956/wellcomeopenres.16178.r35023

**Published:** 2019-04-18

**Authors:** Rada Savic

**Affiliations:** 1 Department of Bioengineering and Therapeutic Sciences, University of California, San Francisco (UCSF), San Francisco, CA, USA

We congratulate the authors on using a well-designed modeling approach to improve our understanding of how exposure to dihydroartemisinin, a critical agent for malaria treatment, is reduced in pregnancy due to increased dihydroartemisinin clearance. This is a particularly relevant contribution, as pregnant women are especially vulnerable to malaria and its consequences. In particular, we appreciate the author’s clear description and thorough approach to the modeling methods.

 We suggest a few clarifications.

 Major Comments:

- Use of allometric scaling results in weight-based dosing recommendations for patients, which can complicate dosing regimens, and at the extreme could lead to lower drug coverage in low weight women and potential toxicity for larger women. We note a relatively small change in objective function value with addition of allometric scaling to the model. Was there a detectable negative correlation between weight and drug concentration in the observed data to suggest lower concentrations in women with greater weights?
- It is interesting that pregnant women had higher parasite biomass, and that pregnancy and parasite biomass had opposing covariate effects on CL/F. Specifically, higher dihydroartemisinin clearance was associated with pregnancy and higher relative bioavailability associated with higher parasite biomass. Can the authors comment on how they distinguished the covariate effects of pregnancy and parasite biomass during modeling (e.g. through evaluation of residual errors, OFV, VPCs, etc)? How did including parasite biomass impact the pregnancy covariate relationship?

Minor Comments:

- Showing the VPC for dihydroartemisinin stratified by pregnancy status may be helpful for the reader.
- Can the authors clarify which model was used for the final parameters listed in Table 2? One option would be to reverse the sentences in the third paragraph on page 7 of the results section as follows: “Final parameter estimates are summarized in Table 2. The full covariate approach investigating the impact of pregnancy confirmed the above covariate relationship, resulting in an increased dihydroartemisinin elimination clearance of 24% in pregnant women, causing a proportional decrease in total drug exposure to dihydroartemisinin (Figure 3)."
- Please include the change in OFV associated with adding the covariate parasite biomass in the text.
- Please reword the first sentence of the results section to improve clarity. As it reads now it could be thought you had 48 pregnant women not 48 women total.
- Please remove the extra “)” at the end of this sentence. “In addition, parasite biomass and ALT at enrolment were found to be significant covariates on the relative bioavailability; 14% increase in relative bioavailability per each increase in the natural logarithm of the parasite biomass, and 2.2% increase in relative bioavailability per each increase in ALT).”

 

 In summary, we thank the authors for contributing to our understanding of artesunate and dihydroartemisinin pharmacokinetics during pregnancy. We appreciate their inclusion of NONMEM code to aid understanding and future efforts investigating this combination.  We agree with their conclusions that more work is needed clarify how differences in pharmacokinetics impact treatment outcomes for pregnant women with malaria who are treated with ACTs, particularly when immunity in the population is low.

I confirm that I have read this submission and believe that I have an appropriate level of expertise to confirm that it is of an acceptable scientific standard.

# Response

**Published:** 2019-12-20

**Authors:** Joel Tarning

**Affiliations:** Mahidol-Oxford Tropical Medicine Research Unit, Thailand

Major Comments:

1. Use of allometric scaling results in weight-based dosing recommendations for patients, which can complicate dosing regimens, and at the extreme could lead to lower drug coverage in low weight women and potential toxicity for larger women. We note a relatively small change in objective function value with addition of allometric scaling to the model. Was there a detectable negative correlation between weight and drug concentration in the observed data to suggest lower concentrations in women with greater weights? 

*Reply: We agree with the point raised above by the reviewer, but adults are commonly treated with the same dose of antimalarial drugs while doses are scaled according to weight when treating children (the same dose of artesunate-mefloquine is recommended for all patients ≥30 kg). The main reason for using allometric scaling was to assure that potentially important body size differences were included in the model before evaluating the effect of pregnancy on pharmacokinetic parameters. We agree that the drop in OFV value was relatively small following addition of allometric scaling (∆OFV = 6.3) but it was still an improved model with no additional degrees of freedom. Retaining allometric scaling to bodyweight was also strengthened by literature data of previous population pharmacokinetic modeling of artesunate and dihydroartemisinin in pregnant women (Kloprogge et al, 2015, Br J Clin Pharmacol). However, there was no statistically significant trend when evaluating post-hoc exposure data vs body weight in the limited data presented here. Even so, no difference was seen in median bodyweights between pregnant and non-pregnant women recruited here, and its therefore very unlikely that the implementation of bodyweight as a covariate would generate biased results.*

2. It is interesting that pregnant women had higher parasite biomass, and that pregnancy and parasite biomass had opposing covariate effects on CL/F. Specifically, higher dihydroartemisinin clearance was associated with pregnancy and higher relative bioavailability associated with higher parasite biomass. Can the authors comment on how they distinguished the covariate effects of pregnancy and parasite biomass during modeling (e.g. through evaluation of residual errors, OFV, VPCs, etc)? How did including parasite biomass impact the pregnancy covariate relationship? 

*Reply: Thank you for this insightful comment. We agree that it can be problematic to distinguish between correlated covariate effects. However, we observed a high degree of variability in parasites biomass within the pregnant and non-pregnant group, enabling us to discriminate between these two covariate effects. Both covariates were significant when evaluated in a stepwise manner. Pregnancy, as a covariate on CL/F, was included first and resulted in 18.9% difference between groups. This covariate relationship did not change substantially when including parasite biomass as a covariate on the relative bioavailability (21.4% difference between groups). To confirm these findings, patients were also divided in a pregnant and non-pregnant group, and parasite biomass evaluated as a covariate in each data set. This resulted in parasite biomass as a significant covariate in both of the separate data sets. This finding is also supported by previous literature, reporting opposite effects of pregnancy and parasite biomass (Kloprogge et al. 2015).*

Minor Comments:

1. Showing the VPC for dihydroartemisinin stratified by pregnancy status may be helpful for the reader. 

*Reply: This is an excellent suggestion, but the relatively small number of patients (and data above the LLOQ) in each group makes a stratified VPC rather uninformative due to large simulated confidence intervals around the visualized percentiles. We therefore opted for pooling all the data when performing the simulation-based diagnostics.*

2. Can the authors clarify which model was used for the final parameters listed in Table 2? One option would be to reverse the sentences in the third paragraph on page 7 of the results section as follows: “Final parameter estimates are summarized in Table 2. The full covariate approach investigating the impact of pregnancy confirmed the above covariate relationship, resulting in an increased dihydroartemisinin elimination clearance of 24% in pregnant women, causing a proportional decrease in total drug exposure to dihydroartemisinin (Figure 3)." 

*Reply: Thank you for this suggestion. Changed accordingly.*

3. Please include the change in OFV associated with adding the covariate parasite biomass in the text. 

*Reply: Thank you for this suggestion. Changed accordingly.*

4. Please reword the first sentence of the results section to improve clarity. As it reads now it could be thought you had 48 pregnant women not 48 women total. 

*Reply: The sentence has been revised for clarity and now reads.*

*“48 women (24 pregnant and 24 non-pregnant patients) completed the pharmacokinetic study (one pregnant woman was lost to follow-up due to moving out of the study area).”*

5. Please remove the extra “)” at the end of this sentence. “In addition, parasite biomass and ALT at enrolment were found to be significant covariates on the relative bioavailability; 14% increase in relative bioavailability per each increase in the natural logarithm of the parasite biomass, and 2.2% increase in relative bioavailability per each increase in ALT).” 

*Reply: Thank you for this suggestion. Changed accordingly.*

# Peer review: Reviewer response for version 1

**DOI:** 10.21956/wellcomeopenres.16178.r35129

**Published:** 2019-04-16

**Authors:** Lawrence Fleckenstein

**Affiliations:** 1 College of Pharmacy, University of Iowa, Iowa City, IA, USA

This manuscript reports on population pharmacokinetic modeling of artesunate and dihydroartemisinin in pregnant and non-pregnant women with uncomplicated *Plasmodium falciparum *malaria in Burkina Faso. Women in their second and third trimester of pregnancy and non-pregnant women, all with uncomplicated *P. falciparum* malaria, were studied. Treatment was a fixed-dose combination of oral artesunate and mefloquine once daily for three days. Frequent blood samples were collected and concentration-time data for artesunate and dihydroartemisinin were analysed simultaneously using nonlinear mixed-effects modelling.The principal finding of this manuscript reveals a 21% higher clearance of dihydroartemisinin in pregnant women compared to non-pregnant controls, resulting in a proportionately lower drug exposure. 

 Comments:

1. The authors note in the Study Design section that the clinical details and results from a non-compartmental pharmacokinetic analysis were previously published (Reference 22). Comparing the current manuscript with reference 22 (Both ClinicalTrials.gov NCT00701961) show an apparent difference in the values for the demographics of the study population. Both studies report 24 subjects studied, yet the values for age, weight and height appear to differ (Table 1, Valea vs. Table 1, Birgersson). Please clarify.
2. The values given for baseline characteristics of the study population (haemoglobin, parasite density) do not appear to be the same for the two studies (Table 1, Valea vs. Table 1, Birgersson). Parasite biomass was found by Birgersson to be a significant covariate on relative bioavailability. Please clarify that parasite biomass numbers are correct, and confirm whether it remains a significant covariate.
3. The laboratory parameters for study participants also appear to differ (Table 2, Valea vs. Table 1, Birgersson). This is particularly relevant because the ALT values reported by Valea appear to fall within the normal range, while the maximum values reported by Birgersson appear to be slightly elevated. In the PK analysis by Birgersson, ALT at enrolment was found to be a significant covariate on relative bioavailability. Can the authors confirm this covariate relationship is correct? The last line of paragraph 2 on page 7 of the Birgersson manuscript is unclear, stating: “…2.2% increase in relative bioavailability per each increase in ALT.” Please clarify.
4. To confirm a disease effect with regard to liver status (ALT) as referred on page 9 in the first paragraph; it would seem to require data from individuals with liver disease. It is not clear that evidence of liver disease is present in the study population. The values reported for ALT appear only slightly elevated for some individuals (perhaps isolated and transient values). AST and bilirubin values appear normal and there is no clinical description of liver disease. Please clarify the discussion with respect to the relationship between ALT and liver disease.
5. It would be useful for the authors to comment further in the discussion section on how the transit-compartment absorption model reconciles with the known mechanism of artesunate absorption.

I confirm that I have read this submission and believe that I have an appropriate level of expertise to confirm that it is of an acceptable scientific standard, however I have significant reservations, as outlined above.

# Response

**Published:** 2019-12-20

**Authors:** Joel Tarning

**Affiliations:** Mahidol-Oxford Tropical Medicine Research Unit, Thailand

1. The authors note in the Study Design section that the clinical details and results from a non-compartmental pharmacokinetic analysis were previously published (Reference 22). Comparing the current manuscript with reference 22 (Both ClinicalTrials.gov NCT00701961) show an apparent difference in the values for the demographics of the study population. Both studies report 24 subjects studied, yet the values for age, weight and height appear to differ (Table 1, Valea vs. Table 1, Birgersson). Please clarify. 

*Reply: Thank you for this very observant comment. The presentation of demographic data is based on the same original data in both papers (Valea et al and Birgersson et al). However, data are presented as median and absolute range in the current manuscript, while data were presented as mean and standard deviation in the paper by Valea et al.*

2. The values given for baseline characteristics of the study population (haemoglobin, parasite density) do not appear to be the same for the two studies (Table 1, Valea vs. Table 1, Birgersson). Parasite biomass was found by Birgersson to be a significant covariate on relative bioavailability. Please clarify that parasite biomass numbers are correct, and confirm whether it remains a significant covariate. 

*Reply: The representation of demographic data is based on the same original data in both papers, but presented differently (see comment 1 above). We can confirm that the parasite biomass numbers are correct, and that this was a significant covariate in the model.*

3. The laboratory parameters for study participants also appear to differ (Table 2, Valea vs. Table 1, Birgersson). This is particularly relevant because the ALT values reported by Valea appear to fall within the normal range, while the maximum values reported by Birgersson appear to be slightly elevated. In the PK analysis by Birgersson, ALT at enrolment was found to be a significant covariate on relative bioavailability. Can the authors confirm this covariate relationship is correct? The last line of paragraph 2 on page 7 of the Birgersson manuscript is unclear, stating: “…2.2% increase in relative bioavailability per each increase in ALT.” Please clarify. 

*Reply: Similar to the above comments, also the laboratory data is based on the same original data in both papers, but presented differently (i.e. median and range vs mean and standard deviation). We can confirm that the demographic and laboratory data is presented according to the original database in the current manuscript. ALT was a significant covariate on the relative bioavailability. We agree that the sentence mentioned above is unclear and we have re-written it to clarify the results.*

*“…2.2% increase in relative bioavailability per each unit (IU) increase in ALT.”*

4. To confirm a disease effect with regard to liver status (ALT) as referred on page 9 in the first paragraph; it would seem to require data from individuals with liver disease. It is not clear that evidence of liver disease is present in the study population. The values reported for ALT appear only slightly elevated for some individuals (perhaps isolated and transient values). AST and bilirubin values appear normal and there is no clinical description of liver disease. Please clarify the discussion with respect to the relationship between ALT and liver disease. 

*Reply: This is a very important comment raised by the reviewer. Both artesunate and dihydroartemisinin are metabolized via liver enzymes; cytochrome P450-family and UGT-enzymes, respectively. It was therefore of interest to evaluate ALT and AST as covariates in the population pharmacokinetic analysis to describe the potential impact of these on different parameters related to metabolism. It was not an objective to evaluate liver disease in this population, but the intention was to characterize the pharmacokinetic data and explain the observed variability. Thus, we do not claim to have characterized the impact of liver disease, but we could still identify a relationship between observed values of ALT in different patients and their estimated relative bioavailability. We agree that this covariate description can be misunderstood on account of using “liver status”, and we have reworded the section to clearly state that there was a relationship found between observed values of ALT and the relative bioavailability.*

5. It would be useful for the authors to comment further in the discussion section on how the transit-compartment absorption model reconciles with the known mechanism of artesunate absorption. 

*Reply: Thank you for this comment. Artesunate is known to have a rapid absorption with short lag-time in the absorption, resulting in detectable concentrations within 15 min of drug administration in healthy volunteers and patients (summarized in Morris et al, 2011). In this paper several different absorption models were evaluated; zero-order, first-order, absorption lag-time, sequential absorption, and a flexible transit compartment model with 1–10 transit compartments. The transit compartment model was superior to all other models tested with an estimated mean transit absorption time of 0.8h. This is in agreement with a previously published population pharmacokinetic model of artesunate in pregnant women (Kloprogge et al 2015). Furthermore, the transit absorption model offers a biologically plausible parameterization of drugs that show varying degrees of lag-time absorption. We have expanded the discussion slightly to clarify this.*
